# Increase of circulating memory B cells after glucocorticoid-induced remission identifies patients at risk of IgG4-related disease relapse

**DOI:** 10.1186/s13075-018-1718-5

**Published:** 2018-10-03

**Authors:** Marco Lanzillotta, Emanuel Della-Torre, Raffaella Milani, Enrica Bozzolo, Emanuele Bozzalla-Cassione, Lucrezia Rovati, Paolo Giorgio Arcidiacono, Stefano Partelli, Massimo Falconi, Fabio Ciceri, Lorenzo Dagna

**Affiliations:** 1 Università Vita-Salute San Raffaele, IRCCS-San Raffaele Scientific Institute, Milan, Italy; 2 Unit of Immunology, Rheumatology, Allergy and Rare Diseases (UnIRAR), IRCCS-San Raffaele Scientific Institute, via Olgettina 60, 20132 Milan, Italy; 3 Unit of Immunohematology and Transfusion Medicine, IRCCS-San Raffaele Scientific Institute, Milan, Italy; 4 Pancreato-Biliary Endoscopy and Endosonography Division, IRCCS-San Raffaele Scientific Institute, Milan, Italy; 5 Division of Pancreatic Surgery, Pancreas Translational and Clinical Research Center, IRCCS-San Raffaele Scientific Institute, Milan, Italy; 6 Hematology and Bone Marrow Transplantation Unit, IRCCS-San Raffaele Scientific Institute, Milan, Italy

**Keywords:** IgG4, IgG4-related disease, B cells, Plasmablasts, Corticosteroid, Glucocorticoid, Therapy, Treatment

## Abstract

**Background:**

Immunoglobulin G4-related disease (IgG4-RD) promptly responds to glucocorticoids but relapses in a considerable fraction of patients. Reliable biomarkers of flare are currently lacking because the pathophysiology of IgG4-RD remains largely elusive. In the present work, we aimed to identify perturbations of B-cell subpopulations that might predict IgG4-RD relapse.

**Methods:**

Thirty patients were treated with glucocorticoids according to international guidelines. Circulating CD19^+^ and CD20^+^ cells, naive B cells, memory B cells, plasmablasts, and plasma cells were measured by flow cytometry at baseline and every 6 months for 2 years after the initiation of corticosteroid therapy.

**Results:**

Patients with active untreated IgG4-RD showed significantly reduced CD19^+^ B cells, CD20^+^ B cells, and naive B cells compared with healthy subjects (*p* < 0.05), but significantly expanded plasmablasts and plasma cells (*p* < 0.01). After 6 months of corticosteroid treatment, all patients achieved clinical improvement. Naive B cells, plasmablasts, and plasma cells significantly decreased compared with disease onset, whereas memory B cells significantly increased compared with baseline (*p* < 0.01). Increase of memory B cells was observed only in patients who relapsed within 2 years of follow-up, however (HR, 12.24; 2.99 to 50.2; *p* = 0.0005). In these patients, the relapse rates at 12 and 24 months were 30% and 100%, respectively. No abnormalities of other B-cell subpopulations at disease onset or after 6 months of glucocorticoid treatment were found to predict IgG4-RD relapse at 2 years.

**Conclusions:**

Increase of circulating memory B cells after 6 months of glucocorticoid treatment might predict IgG4-RD relapse.

## Background

Immunoglobulin G4-related disease (IgG4-RD) is a systemic fibroinflammatory condition characterized by tumorlike expansive lesions and often by abnormal increase of serum IgG4 concentration [1]. Glucocorticoid treatment leads to remission in the majority of patients, but IgG4-RD relapses within 2 years in up to 50% of cases, both during tapering and after withdrawal of corticosteroid therapy [2, 3].

Relapses represent a major clinical problem in the long-term management of patients with IgG4-RD, for several reasons. First, flares carry an additional risk of organ damage and life-threatening complications because they might involve the same organs affected at disease onset or different anatomical sites [3]. Second, relapsing patients are at higher risk of steroid-related adverse effects because they are typically treated with higher cumulative doses of glucocorticoids [3, 4]. Also, preventive follow-up and therapeutic strategies cannot be adopted, because we currently lack reliable biomarkers to identify patients who will relapse and to predict the timing of flares. Indeed, peripheral blood eosinophilia, elevated serum immunoglobulin E (IgE), and IgG4 at disease onset have traditionally been proposed as predictors of recurrence, but a better understanding of the natural history of IgG4-RD has gradually unveiled the shortcomings of these biomarkers [1, 2, 5, 6]. The majority of patients with IgG4-RD do not in fact show increased eosinophils counts or serum IgE levels at the time of diagnosis, and most of the data regarding the value of IgG4 levels in predicting disease relapse failed to demonstrate any definitive associations in different study cohorts [7, 8]. Reliable predictors of IgG4-RD flare are therefore still missing, and their identification requires a better comprehension of the pathophysiological mechanisms that initiate and sustain disease activity.

Recent observations suggest that the B-cell compartment might be central to IgG4-RD pathogenesis. Indeed, B-cell depletion therapy with rituximab induces prompt clinical responses [9]. Plasmablasts are oligoclonally expanded in patients with active disease [10, 11], disappear with clinical improvement [10, 12], and increase with disease relapse [10, 11]. Given this emerging role of B lymphocytes in the pathogenesis of IgG4-RD, in the present study we aimed to identify alterations of B-cell subsets that might predict IgG4-RD flare after initial response to glucocorticoid treatment.

## Methods

### Patients, disease activity assessment, and treatment

Thirty patients with active untreated IgG4-RD referred to our tertiary care center between September 2014 and December 2016 were consecutively included in the present prospective monocentric study. IgG4-RD was diagnosed according to the consensus statement on the pathology of IgG4-RD and the comprehensive diagnostic criteria for IgG4-RD [13, 14]. Patients with pancreatic involvement who did not undergo histological confirmation were diagnosed with “definite” IgG4-RD according to the international consensus diagnostic criteria for autoimmune pancreatitis [15]. All patients were treated with oral prednisone at an initial dose of 0.6–1 mg/kg for 1 month. Prednisone was then tapered in accordance with international guidelines and withdrawn whenever possible after 4–6 months [3]. IgG4-RD activity was assessed by means of the immunoglobulin G4-related disease responder index (IgG4-RD RI) [16]. Active disease was defined by an IgG4-RD RI ≥ 3. Complete response vs. disease remission was defined by an IgG4-RD RI < 3 in the presence or absence of concomitant corticosteroid treatment, respectively. A reduction of the IgG4-RD RI but still with a total score ≥ 3 was considered a partial response to treatment. Relapses were defined as increases in the IgG4-RD RI ≥ 2 and/or the need for the reinstitution of treatment. Blood samples for immunological studies were drawn at baseline and every 6 months for 2 years after the initiation of glucocorticoid treatment. Twenty healthy age- and sex-matched subjects were studied as control subjects. All subjects enrolled provided written informed consent for the analyses performed. The study was conducted according to the Declaration of Helsinki and approved as a descriptive noninterventional study by the ethics committee of the San Raffaele Scientific Institute.

### Laboratory and flow cytometric analyses

Laboratory analyses included C-reactive protein (CRP), erythrocyte sedimentation rate (ESR), total serum IgE, total serum immunoglobulin G (IgG), IgG1, IgG2, IgG3, and IgG4 subclasses. Flow cytometry was performed using a Navios cytometer (Beckman Coulter, Brea, CA, USA) on fresh peripheral blood collected in ethylenediaminetetraacetic acid tubes using a lyse-no-wash technique (ammonium chloride) and the following panel of directly conjugated antibodies: CD3-fluorescein isothiocyanate, CD4-ECD, CD8-Pacific Blue, CD19-A700, CD20-allophycocyanin, CD27-phycoerythrin-cyanine 7, CD38-A750, CD45-Krome Orange, CD56-phycoerythrin, CD138-PC5.5 (Beckman Coulter). Naive B cells, memory B cells, plasmablasts, and plasma cells were identified within the CD19^+^ gate as CD19^+^CD20^+^CD27^−^CD38^+^ cells, CD19^+^CD20^+^CD27^+^CD38^−^ cells, CD19^+^CD20^−^CD27^+^CD38^+bright^ cells, and CD19^+^CD20^−^CD38^+^CD138^+^ cells, respectively. Total B cells were identified both as CD19^+^ cells (CD19^+^/side scatter [SSC] within the leukogate) and CD20^+^ cells (CD20^+^/SSC within the leukogate).

### Statistical analysis

Statistical analysis was performed using Prism software 6.0 (GraphPad Software, La Jolla, CA, USA). Normal distribution of continuous variables was assessed with the D’Agostino and Pearson omnibus normality test. Normally distributed variables were compared using Student’s *t* test. Nonnormally distributed variables were compared using the Mann-Whitney *U* test. Follow-up nonnormally distributed variables were compared using the Wilcoxon test. Nonparametric correlations were calculated using Spearman’s correlation. Linear correlations were measured by Pearson’s correlation coefficient. A *p* value < 0.05 was considered statistically significant. Values are presented as median and IQR, unless specified otherwise. Kaplan-Meier curves were used to assess time to relapse. Times to relapse in subgroups were compared using the log-rank test. The HR was computed using the Mantel-Haenszel approach.

## Results

### Distribution of B-cell subpopulations in patients with active untreated IgG4-RD

**Fig. 1:**
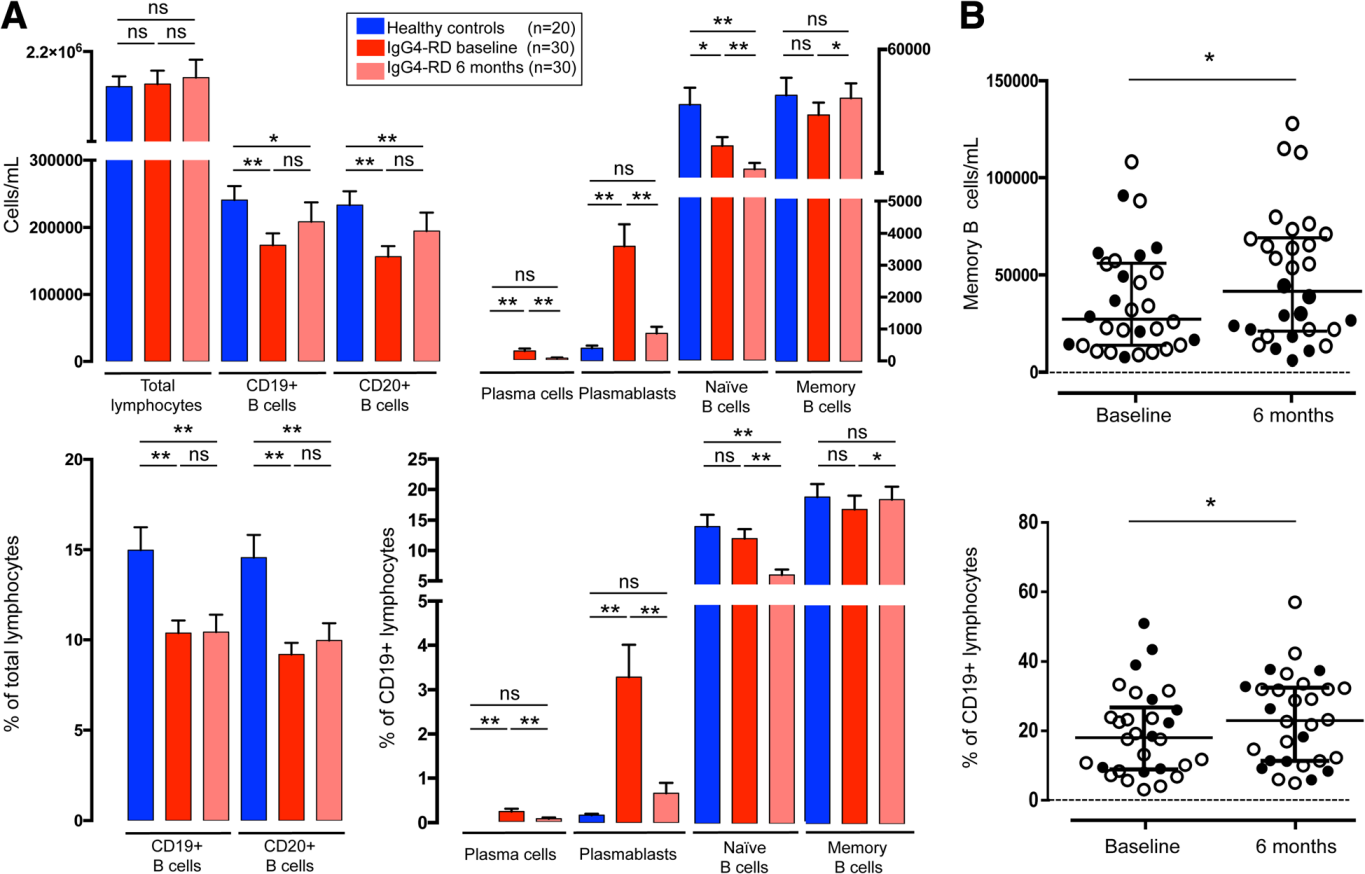
**a** Distribution of B-cell subsets in healthy control subjects and in patients with immunoglobulin G4-related disease (IgG4-RD) at baseline and after 6 months of glucocorticoid treatment in absolute counts and percentage of CD19^+^ B lymphocytes. **b** Memory B cells at baseline and after 6 months of glucocorticoid treatment in absolute counts and as percentage of CD19^+^ B lymphocytes. *Open* and *filled dots* indicate patients showing memory B-cell increase and decrease after treatment, respectively. Results are expressed as mean ± SEM. * *p* < 0.05; ** *p* < 0.01. *ns* Not statistically significant

Thirty patients with active untreated IgG4-RD were included in this prospective study. Clinical, serological, and immunological features of the study cohort are summarized in Table 1. The distribution of B-cell subpopulations in absolute numbers and percentage of CD19^+^ B lymphocytes is shown in Fig. 1a. At baseline, total lymphocyte count in patients with IgG4-RD was comparable to that of healthy subjects. Flow cytometric analysis revealed a significant CD19^+^ and CD20^+^ B-cell lymphopenia in patients with IgG4-RD, both in absolute counts and in percentage of total lymphocytes compared with healthy control subjects (*p* < 0.05). Absolute number of naive B cells—but not the percentage over total CD19^+^ lymphocytes—was also significantly reduced in patients with IgG4-RD compared with healthy subjects (*p* < 0.01). The levels of memory B cells were comparable between patients with IgG4-RD and healthy individuals, both in absolute numbers and in percentage of CD19^+^ B cells. Absolute plasmablast counts and their percentage over total CD19^+^ B cells were significantly increased in patients with IgG4-RD compared with healthy control subjects (*p* < 0.0001). Circulating plasma cells were detected in 16 (53.3%) patients with IgG4-RD and in none of the healthy individuals.

**Table 1:** Clinical, serological, and immunological features of the patient cohort at baseline and after treatment with glucocorticoids

	Patients with IgG4-RD before GC (*n* = 30)	Healthy control subjects (*n* = 20)	*p* Value	Patients with IgG4-RD after GC (*n* = 30)	*p* Value
Definite IgG4-RD, *n* (%)	29 (97%)				
Probable IgG4-RD, *n* (%)	1 (3%)				
Possible IgG4-RD, *n* (%)	0 (0%)				
Age, yr, median	70 (58–73)	54 (46–65)	0.005		
Male sex, *n* (%)	23 (77%)	12 (60%)			
ESR (0–20 mm/h)	18 (10–35)				
CRP (< 6 mg/L)	5 (2–6)				
IgG4-RD RI (0–3)	6 (6–9)			2 (1–2.25)	0.0001
Serum IgG4 (< 135 mg/dl)	313 (206–507)			191 (87–230)	0.0001
CD19^+^ B cells (cells/ml)	162,000 (105,750–217,750)	236,000 (200,000–299,000)	0.0002	163,500 (100,750–233,500)	0.131
CD20^+^ B cells (cells/ml)	144,500 (93,000–201,700)	224,000 (199,000–279,000)	0.0001	150,500 (85,500–226,250)	0.1
Naive B cells (cells/ml)	15,120 (8895–29,140)	23,810 (17,930–54,020)	0.01	7485 (4195–14,018)	0.0001
Percentage of CD19^+^ B cells	10.55 (7.94–15.49)	13.02 (7.89–19.39)	0.35	4.78 (3.14–8.33)	0.0001
Memory B cells (cells/ml)	26,475 (13,040–55,450)	37,170 (21,900–57,190)	0.25	41,800 (21,148–69,435)	0.026
Percentage of CD19^+^ B cells	18.5 (9.26–27.31)	16.60 (9.18–26.34)	0.62	22.89 (11.14–32.50)	0.028
Plasmablasts (cells/ml)	2515 (1023–5550)	340 (170–600)	0.0001	270 (210–1198)	0.0001
Percentage of CD19^+^ B cells	1.25 (0.6–4.51)	0.19 (0.05–0.29)	0.0001	0.23 (0.1–0.79)	0.0001
Plasma cells (cells/ml)^a^	278 (0–1332)	0 (0–0)	0.0005	55 (0–423)	0.0006
Percentage of CD19^+^ B cells^a^	0.23 (0–1.27)	0 (0–0)	0.0001	0.07 (0–0.64)	0.0008
Organ involvement, *n* (%)					
Pancreas	20 (66%)				
Aorta and retroperitoneum	7 (23.3%)				
Lymph nodes	5 (16.6%)				
Biliary tree	5 (16.6%)				
Salivary glands	2 (6.6%)				
Lacrimal glands	2 (6.6%)				
Lung	2 (6.6%)				
Orbit	1 (3.3%)				
Nasal sinuses	1 (3.3%)				
Meninges	1 (3%)				
Kidney	1 (3.3%)				

*Abbreviations*: *CRP* C-reactive protein, *ESR* Erythrocyte sedimentation rate, *IgG4-RD RI* IgG4-related disease responder index, *GC* Glucocorticoids

Results are expressed as median (IQR), except where indicated otherwise

^a^ Results expressed as mean (range)

### Effects of glucocorticoids on B-cell subpopulations in patients with IgG4-RD

All patients were treated with glucocorticoids according to international guidelines (*see* the “Methods” section above), and B-cell subpopulations were studied after 6 months of treatment (Table 1) [3]. At that time point, clinical improvement was observed in all patients, with an IgG4-RD RI that decreased from a median baseline value of 6 (IQR, 6–9) to 2 (IQR, 1–2.25) (paired *p* < 0.05). Seven patients achieved partial response, 20 patients achieved complete response, and 3 patients achieved disease remission. The median daily dose of prednisone at the time of follow-up analysis was 5 mg (range, 0–10 mg).

Effects of corticosteroids on B-cell subpopulations are reported in Fig. 1a. Total lymphocytes, CD19^+^, and CD20^+^ B-cell counts were not affected by glucocorticoids. Naive B cells, plasmablasts, and plasma cells significantly decreased compared with baseline, both in absolute numbers and as a percentage of total CD19^+^ B cells (paired *p* < 0.01 for all comparisons). The absolute number of memory B cells and their percentage over CD19^+^ B lymphocytes significantly increased with disease improvement (paired *p* < 0.05).

### Predictors of IgG4-RD relapse at baseline

In order to evaluate differences within the B-cell compartment of relapsing and nonrelapsing patients with IgG4-RD, we further focused our analysis on 15 subjects followed for at least 24 months. Twenty-four months represented a reliable follow-up to identify relapsing and nonrelapsing patients, because most IgG4-RD cases are known to recur within 2 years after initiation of immunosuppressive therapies [2]. The remaining 15 patients of the study cohort were not included in the analysis, because they did not have an adequate follow-up, and we could not classify them as either relapsers or nonrelapsers.

Ten of the fifteen patients followed for 2 years relapsed, on average, 18 months (range, 8–24) after the diagnosis, one on 10 mg of daily prednisone, three on 5 mg, and six off glucocorticoids. The median durations of glucocorticoid treatment in relapsing and nonrelapsing patients were 9 months (range, 6–18) and 7 months (range, 4–13), respectively. Clinical and laboratory features of relapsing and nonrelapsing patients are reported in Table 2. Multiorgan involvement was present in seven of ten relapsing and four of five nonrelapsing patients. The pancreas, the biliary tree, and the aorta were affected in five of ten, two of ten, and two of ten relapsing patients and in four of five, one of five, and one of five nonrelapsing patients, respectively. Lymph node involvement was present in two relapsing and two nonrelapsing patients. At baseline, we did not observe any statistically significant difference between the two study groups with respect to the IgG4-RD RI, starting prednisone dose, serum IgE, and IgG4 concentrations (*p* > 0.05 for all comparisons). Eosinophil counts were significantly higher in relapsing patients (*p* < 0.05). In particular, they were elevated in four of ten relapsers (median, 300; range, 200–1800 cell/mm^3^) and normal in all nonrelapsers (median, 200; range, 100–300 cell/mm^3^) (Table 2). Total CD19^+^ cells, CD20^+^ cells, naive B cells, memory B cells, circulating plasmablasts, and plasma cells also did not differ between relapsing and nonrelapsing patients, both in absolute numbers and as a percentage of CD19^+^ B cells (*p* > 0.05 for all comparisons).

**Table 2:** Clinical, serological, and immunological features of relapsing and nonrelapsing patients at baseline and after treatment with glucocorticoids

	Relapsers (*n* = 10)	Nonrelapsers (*n* = 5)	*p* Value
Definite IgG4-RD (%)	9 (90%)	5 (100%)	
Probable IgG4-RD (%)	1 (10%)	0 (0%)	
Possible IgG4-RD (%)	0 (0%)	0 (0%)	
Age, yr, median	69 (60–71)	73 (64–80)	0.13
Male, *n* (%)	9 (90%)	3 (60%)	
Multiorgan involvement (> 1 organ)	7 (70%)	4 (80%)	
Baseline			
ESR (0–20 mm/h)	10 (9–23)	15 (8–20)	0.59
CRP (< 6 mg/L)	5 (4–6.5)	10 (5–46)	0.06
IgG4-RD RI (0–3)	9 (6–9)	12 (9–12)	0.22
Eosinophils (< 300 cell/μl)	300 (300–500)	200 (150–300)	0.034
Serum IgG4 (< 135 mg/dl)	364 (232–1090)	498 (328–947)	0.5
IgE (mU/ml)	308 (2–1488)	733 (271–1554)	0.11
Prednisone dose (mg/d)	5 (0–5.5)	5 (2.5–5)	0.99
CD19^+^ B cells (cells/ml)	138,500 (97,500–172,500)	144,000 (103,000–162,000)	0.66
CD20^+^ B cells (cells/ml)	114,000 (86,250–150,000)	128,000 (82,000–140,500)	0.57
Naive B cells (cells/ml)	14,170 (9518–24,198)	11,170 (2915–38,650)	0.35
Percentage of CD19^+^ B cells	11.4 (9.5–13.7)	11.52 (2.33–24.37)	0.09
Memory B cells (cells/ml)	20,450 (10,790–36,070)	48,590 (11,305–62,095)	0.44
Percentage of CD19^+^ B cells	15.79 (10.25–23.9)	26.48 (6.9–47.88)	0.67
Plasmablasts (cells/ml)	3280 (985–9868)	5400 (3825–8000)	0.39
Percentage of CD19^+^ B cells	3.26 (0.84–7.8)	3.38 (2.06–4.82)	0.76
Plasma cells (cells/ml)^a^	420 (0–1332)	489 (146–1300)	0.86
Percentage of CD19^+^ B cells^a^	0.37 (0–1.27)	0.27 (0.1–0.49)	0.95
After 6 mo of treatment			
ESR (0–20 mm/h)	5 (3–21)	9 (8–20)	0.29
CRP (< 6 mg/L)	2 (1–2.25)	2 (1.5–4)	0.47
IgG4-RD RI (0–3)	2.5 (1.75–3.25)	2 (2–2.5)	0.62
Eosinophils (< 300 cell/μl)	200 (100–325)	100 (100–200)	0.37
Serum IgG4 (< 135 mg/dl)	182.5 (107–729)	257 (211–406)	0.42
IgE (mU/ml)	107 (2–299)	425 (384–466)	0.13
Prednisone dose (mg/d)	5 (0–5.62)	5 (2.5–5)	0.99
CD19^+^ B cells (cells/ml)	174,500 (93,750–222,250)	128,000 (64,500–157,500)	0.2
CD20^+^ B cells (cells/ml)	165,000 (84,750–208,500)	128,000 (52,500–154,000)	0.24
Naive B cells (cells/ml)	7860 (3988–13,585)	7380 (2950–15,460)	0.8
Percentage of CD19^+^ B cells	3.51 (2.57–4.13)	9.27 (4.16–15.73)	0.1
Memory B cells (cells/ml)	60,540 (21,148–75,428)	18,360 (9045–34,650)	0.05
Percentage of CD19^+^ B cells	27.46 (19.06–34.9)	24.19 (6.43–37.65)	0.89
Plasmablasts (cells/ml)	355 (138–1263)	1310 (565–3350)	0.07
Percentage of CD19^+^ B cells	0.27 (0.07–0.53)	0.88 (0.36–5.3)	0.03
Plasma cells (cells/ml)^a^	56 (0–333)	143 (0–423)	0.22
Percentage of CD19^+^ B cells^a^	0.05 (0–0.32)	0.19 (0–0.53)	0.16

*Abbreviations*: *CRP* C-reactive protein; *ESR* erythrocyte sedimentation rate; *IgG4-RD RI* IgG4-Related Disease Responder Index

Results are expressed as median (IQR), except where indicated otherwise

^a^ Results expressed as mean (range)

### Predictors of IgG4-RD relapse after glucocorticoid treatment

After 6 months of glucocorticoid treatment, all 15 patients experienced clinical improvement, with an IgG4-RD RI that decreased from a median baseline value of 9 (IQR, 6–9) to 2.5 (IQR, 1.75–3.25) in relapsing patients, and from a median baseline value of 12 (IQR, 9–12) to 2 (IQR, 2–2.5) in nonrelapsing patients. In particular, five of ten relapsing patients achieved partial response, three of ten achieved complete response, and two of ten achieved disease remission. One of five nonrelapsing patients achieved partial response, three of five achieved complete response, and one of five achieved disease remission. The median daily dose of prednisone at the time of follow-up was 5 mg, both in relapsing patients (range, 0–10 mg) and in nonrelapsing patients (range, 0–5 mg) (*p* = 0.99). The levels of ESR, CRP, eosinophils, and serum IgE and IgG4 were comparable between the two study groups (*p* > 0.05 for all comparisons).

**Fig. 2:**
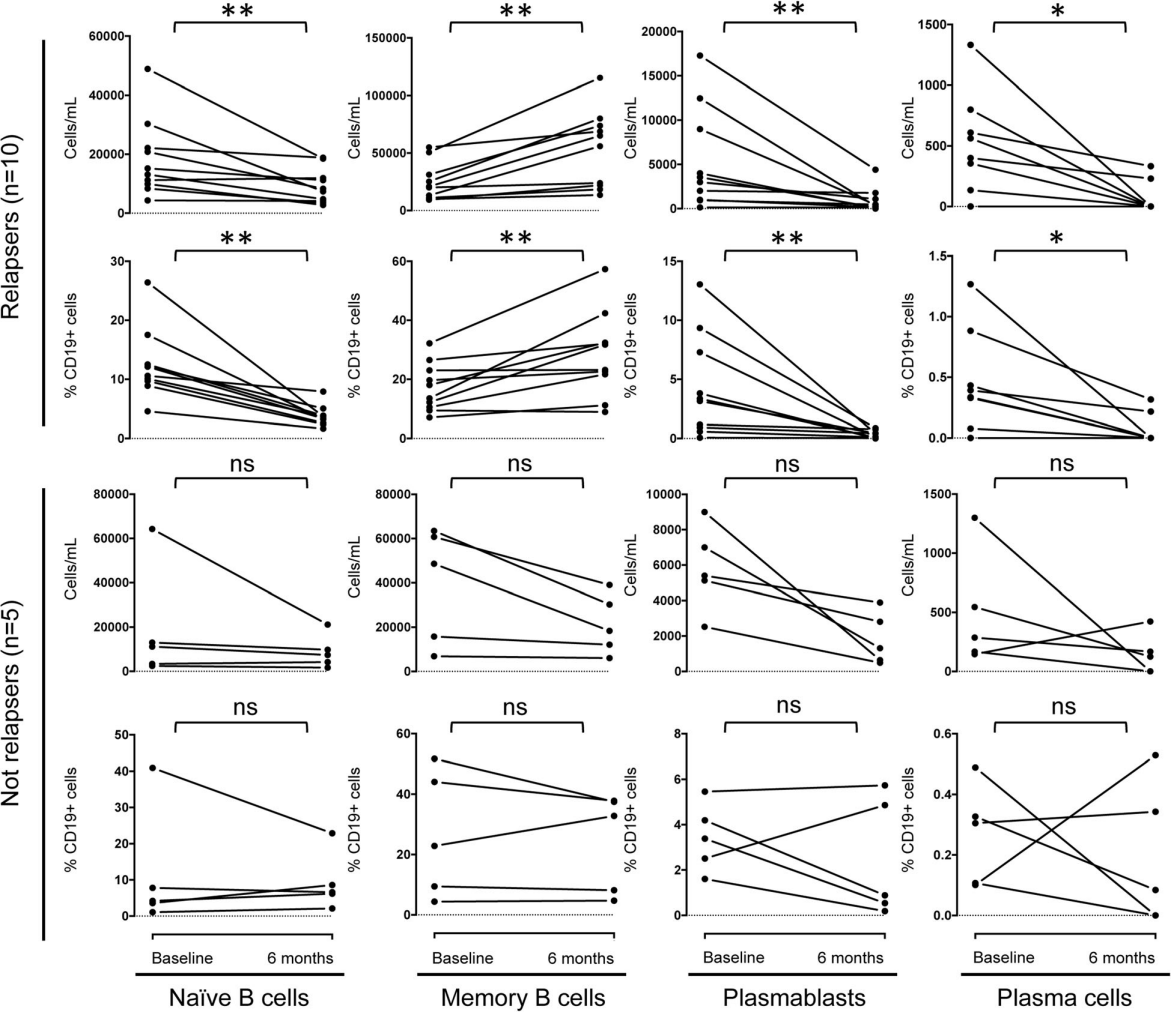
B-cell subset modifications after treatment with glucocorticoids in relapsing and nonrelapsing patients with immunoglobulin G4-related disease, in absolute counts and as percentage of CD19^+^ B lymphocytes. * Paired *p* value < 0.05; ** paired *p* value < 0.01. *ns* Not statistically significant

Total CD19^+^ cell and CD20^+^ cell count did not differ between relapsing and nonrelapsing patients (*p* > 0.05). Similarly, the absolute numbers of naive B cells and plasma cells, as well as their percentage over total CD19^+^ B cells, were comparable between the two study groups (*p* > 0.05 for all comparisons) (Table 2). Conversely, memory B cells and circulating plasmablasts were significantly higher in relapsing and nonrelapsing patients, respectively. Yet, although absolute counts of naive B cells, plasmablasts, and plasma cells uniformly decreased in all patients compared with baseline values, memory B cells decreased only in nonrelapsing patients and increased in all relapsing patients (Fig. 2). A similar trend of the memory B cell/CD19^+^ B cell ratio—namely, a decrease in nonrelapsing patients and an increase in relapsing patients—was observed in nine of ten relapsing patients and in three of five nonrelapsing patients (Fig. 2).

**Fig. 3:**
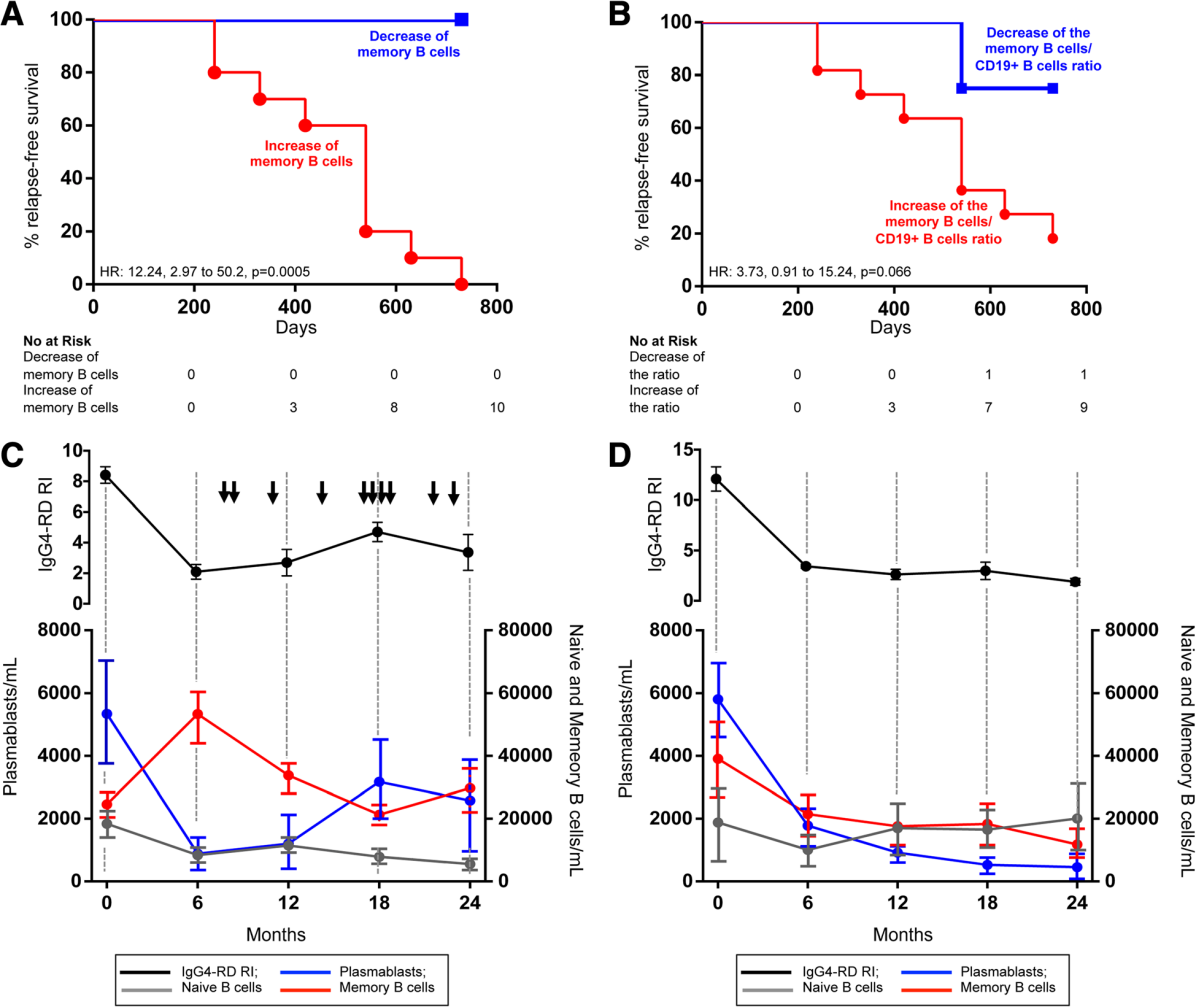
Kaplan-Meier plots of the risk of immunoglobulin G4-related disease (IgG4-RD) relapse in patients showing memory B-cell increase or decrease after 6 months of glucocorticoid therapy in absolute counts (**a**) and as percentage of CD19^+^ B lymphocytes (**b**). Two-year time course of naive B cells, memory B cells, circulating plasmablasts, and immunoglobulin G4-related disease responder index (IgG4-RD RI) in relapsing (**c**) and nonrelapsing (**d**) patients with IgG4-RD. *Arrows* indicate IgG4-RD flares. Results are presented as mean ± SEM

To further explore the relationship between this opposite behavior of memory B cells in relapsing and nonrelapsing subjects, we performed log-rank survival analysis and confirmed a significantly higher relapse rate among patients with an increase of absolute memory B-cell counts after corticosteroid treatment (HR, 12.24; 2.99 to 50.2; *p* = 0.0005) (Fig. 3a). Relapse rate was also higher in patients showing an increased memory B cell/CD19^+^ B cell ratio after treatment, but this association did not reach statistical significance (HR, 3.73; 0.91 to 15.24; *p* = 0.066) (Fig. 3b). In particular, the increase of absolute memory B-cell counts after 6 months of therapy was associated with relapse rates of 30% at 12 months and 100% at 24 months. The increase of the memory B cell/CD19^+^ B cell ratio was associated with relapse rates of 30% at 12 months and 90% at 24 months. Conversely, the relapse rates of patients showing memory B-cell decrease in absolute counts and as a percentage of CD19^+^ B lymphocytes were 0% and 10%, respectively, at 24 months. A similar opposite trend of memory B cells after corticosteroid treatment was also observed in the remaining 15 patients of the study cohort, but they were not included in the analysis, because their follow-up period was not long enough to identify relapsing and nonrelapsing subjects (Fig. 1b).

Finally, serial measurement of memory B cells and plasmablasts in the ten relapsing patients performed every 6 months until flare showed that disease recurrence was anticipated by a progressive reduction of memory B cells and by a parallel increase of circulating plasmablasts (Fig. 3c). This phenomenon was not observed in nonrelapsing patients, where both memory B cell and plasmablast levels measured every 6 months for 2 years remained comparable to those observed after 6 months of glucocorticoid treatment (or further decreased) (Fig. 3d). Naive B cells did not show any significantly different variation over time between relapsing and nonrelapsing patients (Fig. 3c and d).

## Discussion

The identification of reliable biomarkers for predicting disease flare has been identified as a clinical and research priority in the consensus statement on the treatment and management of IgG4-RD [3]. Indeed, although there is unanimous agreement about the strategies to induce IgG4-RD remission, expert opinions still diverge about how to maintain IgG4-RD response through tailored follow-up and preventive interventions [3].

We investigated B-cell subsets in patients with IgG4-RD before and after standardized corticosteroid treatment, seeking potential biomarkers of disease recurrence, and we observed that an increase in memory B-cell counts after glucocorticoid-induced remission predicted relapse at 2 years. This novel finding may provide clinicians with a tool to reliably identify patients at risk of flare, because it stems from the analysis of B-cell populations that have been causally linked to IgG4-RD [10, 17, 18]. Vice versa, previously reported biomarkers of relapse, such as peripheral blood eosinophilia and serum IgE and IgG4 elevation at baseline, can be considered just bona fide surrogates of disease activity, because their direct involvement in IgG4-RD pathogenesis remains unclear [5, 6]. In addition, relevant shortcomings complicate the use of these biomarkers for preventive follow-up and treatment approaches for the following reasons: (1) cutoff values to identify relapsing and nonrelapsing patients are difficult to establish; (2) the timing of disease relapse remains unpredictable; and (3) as confirmed in the present study, not every patient who ultimately flares shows elevated eosinophils, serum IgE, or IgG4 at disease onset [7, 19]. Conversely, memory B-cell increase after glucocorticoid-induced remission clearly differentiated patients relapsing within 2 years of follow-up from nonrelapsing patients.

Memory B cells represent a heterogeneous group of antigen-experienced B lymphocytes that exhibit a low proliferation rate in physiological conditions but rapidly expand in response to previously encountered invading organisms. Different lymphocyte subsets with opposing functions are now known to be part of the memory B-cell compartment, and their involvement in human autoimmune diseases has been studied extensively [20]. IgD^+^CD27^+^ nonswitched memory B cells with anti-inflammatory properties, for instance, are reduced in systemic lupus erythematosus (SLE) and reconstitute after immunosuppressive treatment [20, 21]. Conversely, proinflammatory IgM^−^IgD^−^CD27^+^ switched and IgD^−^CD27^−^ “double-negative” memory B cells are increased in patients with SLE and rheumatoid arthritis, and their levels correlate with disease activity [20, 22]. In IgG4-RD, increases of IgG4^+^ memory B cells and a decrease of IgM^+^IgD^+^ memory B cells and of IgG1^+^ memory B cells were recently described by Heeringa and colleagues, but a clear correlation with IgG4-RD activity was not established [23]. In this sense, although we currently ignore which memory B-cell subset expands following glucocorticoid therapy in relapsing patients with IgG4-RD, our work is the first, to the best of our knowledge, to show an association between memory B cells and IgG4-RD activity. In particular, we hypothesize that an imbalance between anti-inflammatory and proinflammatory memory B-cell subpopulations might be responsible for disease remission and disease recurrence, respectively.

Similarly, although we currently ignore the mechanisms that drive memory B-cell expansion prior to flare, it is reasonable to think that pathogenic B-cell clones within the memory B-cell compartment might bear an increased resistance to immunosuppressive therapy. Indeed, early repopulation of memory B cells after rituximab therapy has already been associated with relapse in autoimmune disorders such as myasthenia gravis, rheumatoid arthritis, and SLE, thus suggesting that the memory B-cell compartment might act as a reservoir for autoreactive clones of antibody-secreting plasmabasts/plasma cells [24–26]. The notion of pathogenic memory B cells driving disease flare is further supported by the gradual reduction of memory B-cell counts and by the concomitant expansion of circulating plasmablasts that we observed in our cohort prior to IgG4-RD relapse.

Our results show significant points of strength but also have some limitations. First, this is the first study, to our knowledge, that correlates the risk of IgG4-RD relapse with cellular biomarkers of IgG4-RD activity. In addition, the present work has been carried out on one of the largest single-center cohorts of patients with IgG4-RD, an aspect that ensured uniform inclusion criteria and treatment [2]. Furthermore, the relapse rate that we observed in our patient population corresponds to that reported in other IgG4-RD cohorts, indicating that a 2-year follow-up period was an adequate time frame for addressing the primary aim of our study [2–28]. Despite a thorough flow cytometric analysis, however, we did not investigate additional B-cell subsets within the naive and memory compartments that might have varied during the disease course. Further molecular and clonal characterization of memory B-cell subtypes at different stages of disease activity, in fact, could have offered a better understanding of their involvement in the pathogenesis of IgG4-RD. We also recognize that a larger study population might have provided more robust results. However, a multicenter study could have generated biases in the cell population analysis and in patient evaluation. Also, half of our cohort did not have a long-enough follow-up period—namely, 2 years—to reliably classify relapsing and nonrelapsing subjects and was therefore excluded from the analysis.

## Conclusions

To the best of our knowledge, this is the first study assessing B-lymphocyte subpopulations as biomarkers of relapse in IgG4-RD. Our results suggest that memory B-cell increase after 6 months of standardized glucocorticoid therapy may represent a useful tool for identifying patients at risk of flare, regardless of their organ involvement, clinical presentation, and serological status at disease onset, as well as of the duration of corticosteroid treatment. Careful evaluation of the memory B-cell compartment and of its perturbations might therefore be of value for future mechanistic, interventional, and observational studies on IgG4-RD.

## Abbreviations

CRP: C-reactive protein
ESR: Erythrocyte sedimentation rate
IgE: Immunoglobulin E
IgG4-RD RI: Immunoglobulin G4-related disease responder index
IgG4-RD: Immunoglobulin G4-related disease
SLE: Systemic lupus erythematosus
